# Circulating Endothelial Cells in Patients with Venous Thromboembolism and Myeloproliferative Neoplasms

**DOI:** 10.1371/journal.pone.0081574

**Published:** 2013-12-05

**Authors:** Cláudia Torres, Ana Mafalda Fonseca, Magdalena Leander, Rui Matos, Sara Morais, Manuel Campos, Margarida Lima

**Affiliations:** 1 Laboratório de Citometria, Serviço de Hematologia Clínica, Hospital de Santo António (HSA), Centro Hospitalar do Porto (CHP), Porto, Portugal; 2 Unidade Multidisciplinar de Investigação Biomédica (UMIB), Instituto de Ciências Biomédicas Abel Salazar da Universidade do Porto (ICBAS/UP), Porto, Portugal; 3 CICS-UBI-Centro de Investigação em Ciências da Saúde, Universidade da Beira Interior, Covilhã, Portugal; 4 Secção de Trombose e Hemostase, Serviço de Hematologia Clínica, Hospital de Santo António (HSA), Centro Hospitalar do Porto (CHP), Porto, Portugal; University of Padua, Italy

## Abstract

**Background:**

Circulating endothelial cells (CEC) may be a biomarker of vascular injury and pro-thrombotic tendency, while circulating endothelial progenitor cells (CEP) may be an indicator for angiogenesis and vascular remodelling. However, there is not a universally accepted standardized protocol to identify and quantify these cells and its clinical relevancy remains to be established.

**Objectives:**

To quantify CEC and CEP in patients with venous thromboembolism (VTE) and with myeloproliferative neoplasms (MPN), to characterize the CEC for the expression of activation (CD54, CD62E) and procoagulant (CD142) markers and to investigate whether they correlate with other clinical and laboratory data.

**Patients and Methods:**

Sixteen patients with VTE, 17 patients with MPN and 20 healthy individuals were studied. The CEC and CEP were quantified and characterized in the blood using flow cytometry, and the demographic, clinical and laboratory data were obtained from hospital records.

**Results:**

We found the CEC counts were higher in both patient groups as compared to controls, whereas increased numbers of CEP were found only in patients with MPN. In addition, all disease groups had higher numbers of CD62E+ CEC as compared to controls, whereas only patients with VTE had increased numbers of CD142+ and CD54+ CEC. Moreover, the numbers of total and CD62+ CEC correlated positively with the white blood cells (WBC) counts in both groups of patients, while the numbers of CEP correlated positively with the WBC counts only in patients with MPN. In addition, in patients with VTE a positive correlation was found between the numbers of CD54+ CEC and the antithrombin levels, as well as between the CD142+ CEC counts and the number of thrombotic events.

**Conclusions:**

Our study suggests that CEC counts may reveal endothelial injury in patients with VTE and MPN and that CEC may express different activation-related phenotypes depending on the disease status.

## Introduction

The vascular endothelium is strategically located at the interface between tissues and blood [1], being composed by endothelial cells (EC) that form the inner lining of blood vessels [2]. Endothelial cells are metabolically active and play a critical role in many physiological processes, including the maintenance of vascular integrity and the generation of an anti-thrombotic surface [3].

When endothelial injury occurs, the vascular surface acts as a prothrombotic environment, the induction of tissue factor (TF, CD142) and other procoagulant molecules on the EC surface being one of the pivotal steps in this process [4]. Endothelial lesion is also accompanied by the expression of adhesion molecules on the EC membrane, including P-Selectin (CD62P), E-Selectin (CD62E), intercellular adhesion molecule type 1 (ICAM-1, CD54) and vascular cell adhesion molecule type 1 (VCAM-1) [1], [5], [6]. These molecules cause leukocyte recruitment and attachment to the EC, suggesting a role in vascular occlusion [6].

Over the last years it has been proposed that circulating endothelial cells (CEC) may reflect endothelial injury, increased numbers of CEC being observed in different pathological conditions [7], [8], [9], [10]. In addition, a bone-marrow derived cell population - the circulating endothelial progenitor cells (CEP) - has been highlighted, and it has been suggested that these cells contribute to vascular repair [11], [12]. Nevertheless, the number of CEC and CEP in the peripheral blood are exquisitely low, those cells representing about 0.01% to 0.0001% of the mononuclear cells [13], and their quantification is not yet standardized. Of the different methods used, flow cytometry seems the most promising, allowing a rapid multiparametric analysis of these cells [11].

Venous thromboembolism (VTE) is a chronic vascular disease with an average incidence of 117 cases per 100.000 individuals/year [14], which manifests by thrombus formation in the venous system and usually occurs in the legs or as pulmonary embolism [15], [16]. The known risk factors for VTE, that can be genetic and/or acquired, influence the stasis and the hypercoagulability [15]. The genetic risk factors known to be associated with inherited thrombophilia include the gain (e.g. factor V Leiden and prothrombin 20210A mutations) or the loss (i.e., deficiencies in the coagulation inhibitors, antithrombin, protein C and protein S) of coagulation function [17]. Acquired risk factors, such as age, surgery, trauma, immobilization, cancer, pregnancy and the puerperium are useful for estimating the risk of VTE [18]. Nevertheless, they provide little insight into the mechanisms initiating VTE [19], which still needs to be clarified, namely concerning the interaction between the EC and constituents of the blood. [20]

Essential thrombocythaemia (ET) and polycythaemia vera (PV) are myeloproliferative neoplasms (MPN) whose clinical course is mainly characterized by an increased incidence of vascular complications and a tendency to progress into myelofibrosis or acute myeloid leukaemia [21], [22]. Several factors are involved in the pathogenesis of thrombosis occurring in MPN, including patient-related, such as the age, previous thrombotic events (TE), and disease-related (i.e. the presence of JAK2V617F mutation and the baseline leukocyte count) factors, some of which have been used to stratify patients into low and high risk categories and to define treatment strategies [23], [24], [25].

Based on the assumption that CEC and CEP are potential biomarkers for monitoring the vascular integrity and regenerative ability, respectively, we hypothesized that these EC populations have a different expression in the blood of patients with increased risk of thrombosis as compared to healthy individuals. Therefore, we decided to quantify and characterize these cells by flow cytometry in the blood of patients with VTE and patients with MPN, either ET or PV. We also searched for possible correlations between CEC and CEP counts, as well as the CEC expression of activation-induced adhesion molecules and procoagulant markers, with the number of TE, laboratory data and other thrombosis risk factors.

## Materials and Methods

### Ethics Statement

The study was approved by the Ethical Committee of the Centro Hospitalar do Porto and all participants gave written informed consent.

### Study design

A total of 33 adult patients (16 patients with VTE and 17 patients with MPN) recruited from the Clinical Haematology consultation from the Hospital de Santo António, Centro Hospitalar do Porto (Porto, Portugal) were studied. The CEC and CEP were quantified and characterized in the peripheral blood using flow cytometry, and the demographic, clinical and laboratory data were obtained from the hospital records. The results were compared with those obtained in 20 healthy individuals (benevolent blood donors), recruited from the hospital blood bank.

### Subjects

The VTE group consisted of 16 patients, 12 females and 4 males, with a median age of 38 years (range: 22–65 years) who have had at least one venous TE and that have been selected for thrombophilia testing. Venous thromboembolism was defined as deep vein thrombosis, pulmonary embolism or objectively documented thrombosis of deep veins elsewhere in the body [15], which could be unprovoked, i.e. TE occurred without a known risk factors, or manifesting after exposure to triggering circumstances such as surgery, trauma and pregnancy [26]. Criteria for thrombophilia testing were the first thrombosis occurring before age of 50 years, family history of thromboembolism and thrombosis in unusual sites (e.g. cerebral, visceral and upper extremity veins) [27]. The MPN group included 9 patients with ET (7 females and 2 males) and 8 patients with PV (1 female and 7 males), who fulfilled the criteria for the diagnosis, as defined by the World Health Organization in 2008 [21], [22]. The median age of the patients with MPN was 64 years, ranging from 32 to 83 years (ET: 57 years, ranging from 32 to 83 years; PV: 64.5 years, ranging from 59 to 82 years). Patients with VTE and MPN having less than a month over the last TE, or having an acute coronary disease were excluded. Other exclusion criteria were the presence of acute inflammatory processes, active cancers, autoimmune diseases, diabetes *mellitu*s, hepatic and renal dysfunction; patients that had been hospitalized or have had puerperium during the month before the study were also excluded.

Twenty benevolent blood donors, 10 females and 10 males, with a median age of 49.5 years (range: 34–64 years) were used as controls. Blood donors at the first blood donation or that have had recent diseases (<3 months) were excluded.

### Clinical and laboratory data

Besides demographic data, we also recorded the number and location of the TE, as well as the blood counts in all participants. For patients with VTE, the existence of familiar history of TE, personal obstetric events associated with increased risk for thrombosis and other parameters tested in the routine workflow for the screening of thrombophilia, such as the fibrinogen, homocysteine, D-dimers, factor VIII, protein S, protein C and antithrombin (AT) plasma levels, factor V Leiden, prothrombin G20210A and anti-phospholipid antibodies were also registered. Patients with MPN were investigated for the presence of the JAK2 mutation.

### Blood sample collection

Peripheral blood samples used for flow cytometry were collected into a 3 ml tube containing tri-potassium ethylene-diamine-tetra-acetic acid (K_3_-EDTA). In order to avoid the artificial increase in CEC due to venipuncture, the first two millilitres of blood were discarded.

### Flow cytometry studies

#### Technical procedure

Cell staining was performed using a single platform lyse-no-wash direct immunofluorescence technique. Briefly, two hundred microliters of whole blood were incubated for 15 minutes, at room temperature, with two different combinations of monoclonal antibodies (mAb) conjugated with different fluorochromes (Table 1). In order to quantify CEC and CEP, the first staining (anti-CD62E FITC/anti-CD146 PE/anti-CD45 PerCP/anti-CD133 APC) was performed in fluorescent beads containing tubes prepared for absolute cell counting (BD Trucount™ tubes; Becton Dickinson, San Jose, CA) (BD), whereas the second staining (anti-CD142 FITC/anti-CD146 PE/anti-CD45 PerCP/anti-CD54 APC) was done using normal 12×75 mm polypropylene tubes (BD). After incubation, red cell lysis and cell fixation were performed by adding 1 ml of pre-diluted BD FACS Lysing Solution for another 15 minutes, just following by the addition of 2 ml of phosphate buffered saline (PBS) to minimize the effects of the lysing solution on cell integrity. In order to reduce the background, both solutions were previously filtrated with 0.2 µl filters (Thermo Scientific NALGENE). The flow cytometry acquisition was carried on a FACSCalibur (BD) equipped with blue argon and red diode lasers, after a long cleaning procedure (>50 minutes). The instrument performance was monitored daily using the BD CaliBRITE™ beads. The BD Cell Quest Pro™ software was used for data acquisition. The BD Paint-A-Gate Pro™ (BD) and the Infinicyt™ (Cytognos, Salamanca, Spain) programs were used for data analysis.

**Table 1:** Characteristics of the monoclonal antibodies used in this study.

Cluster designation	Clone	Fluorochromes	Supplier	Specificity	Expression on endothelial cells
CD45	2D1	PerCP	BD Bioscience	Leukocyte Common Antigen (LCA)	Progenitor*
CD54	HA58	APC	BD Pharmingen	Intercellular Adhesion Molecule 1 (ICAM-1)	Activated
CD62E	BBIG-E5 (10C10)	FITC	R&D Systems	E-Selectin	Activated
CD133	AC133	APC	Miltenyi Biotec	Prominin 1 (PROM1)	Progenitor
CD142	VD8	FITC	American Diagnostica	Tissue factor	Activated
CD146	P1H12	PE	BD Pharmingen	Melanoma cell adhesion molecule (MCAM or Mel-CAM)	All

Abbreviations: PerCP, peridin-chlorophyll protein; APC, allophycocyanin; FITC, fluorescein isothiocyanate; PE, phycoerythrin.

* CD45 has been described as being absent on mature endothelial cells and expressed at very low levels on endothelial progenitor cells.

#### Data acquisition and analysis

**Figure 1:**
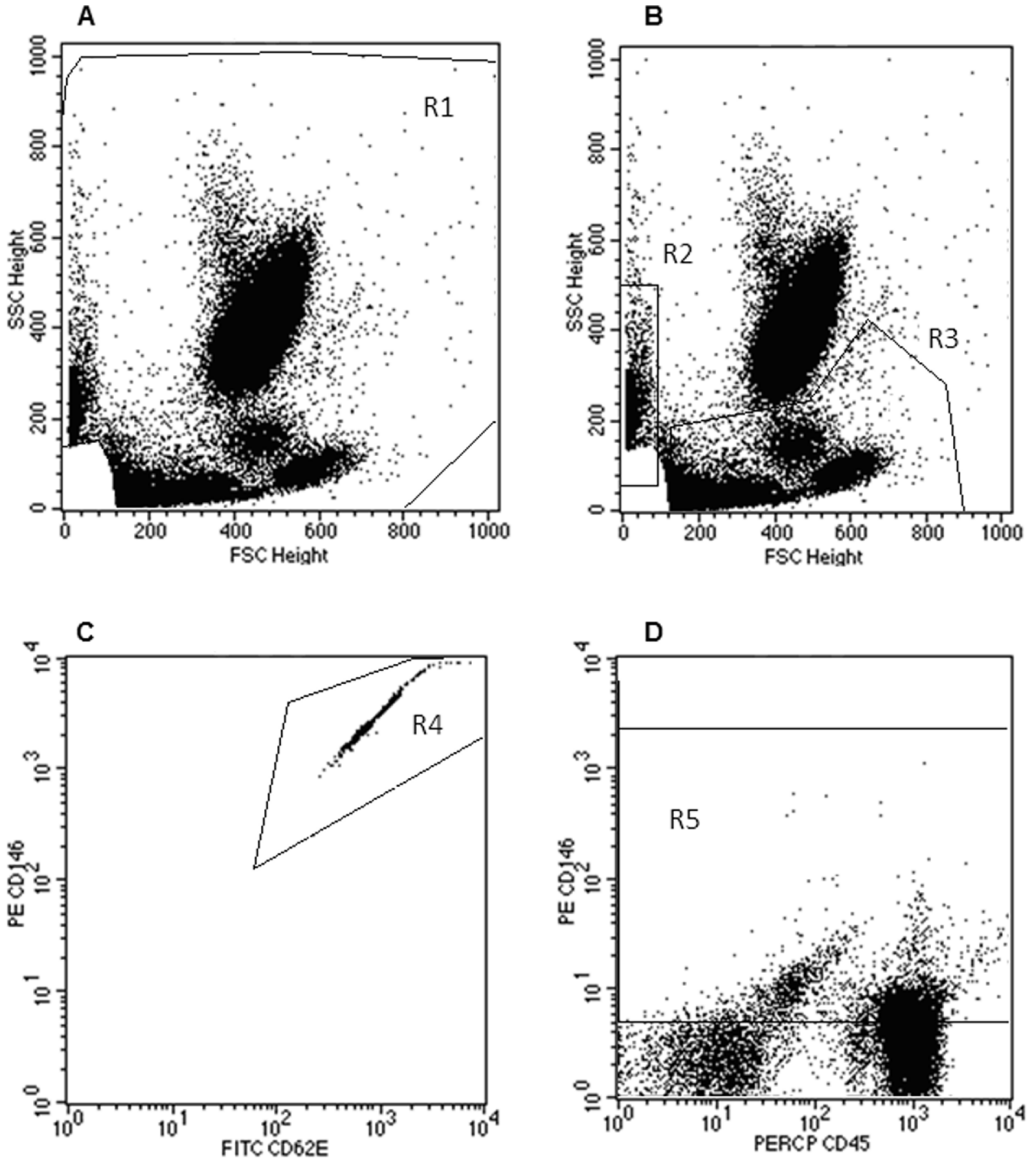
Data acquisition strategy used to identify and quantify the circulating endothelial cells. Our acquisition gating strategy assures the quantification of the circulating endothelial cells (CEC) and endothelial progenitor cells (CEP) without creating enormous FCS2.0 data files. Dot plot **A** illustrates the initial gating based on the FSC/SSC parameters (R1), in order to exclude events with lower FSC/SSC, which comprise mainly debris and platelets; dot plot **B** shows the events within the gate (R1), and includes a gate for Trucount™ beads (R2) and another gate for mononuclear cells (R3); dot plot **C** shows the events within the gate R2 and includes a new gate only for the beads (R4); dot plot **D** shows the events within the gate of mononuclear cells (R3) and contains a new gate only for CD146+ cells, either CD45- or CD45+ (R5). All the events that were inside the R4 (beads) or R5 (CD146+ cells) regions were acquired and stored.

**Figure 2:**
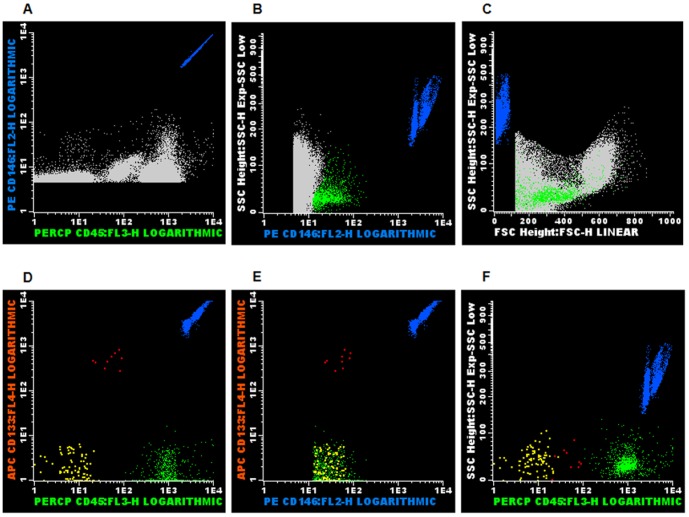
Data analysis strategy used to identify and quantify the circulating endothelial cells. Our data analysis gating strategy assures the identification and quantification of the circulating endothelial cells (CEC) and endothelial progenitor cells (CEP), among stored CD146+ cells. Using the Paint-a-Gate™ or the Infinicyt™ software we first identified the beads (blue dots) in the CD45 vs. CD146 dot plot (A); after that, we selected the total CD146+ events (green dots) (B, for selection of CD146+ cells; C, showing the light scatter properties of the cells); after excluding the CD146- cells (D to F), we finally were obtained the CEC (CD45-CD146+, yellow dots), the CEP (CD45+lowCD146+, red dots), activated lymphocytes (CD45+highCD146+, green dots), as well as the beads (blue dots), being then able to perform CEC and CEP quantification.

The detection, quantification and characterization of CEC and CEP were made using a gating strategy during sample acquisition (Fig. 1), and analysis (Fig. 2). During sample acquisition, a threshold was established on the forward scatter (FSC) in order to exclude debris, except for the BD Trucount™ blood sample tube, in order to include the beads. The CEC and the CEP were identified based on their typical immunophenotype: CD45-CD146+CD133- and CD45+lowCD146+CD133+, respectively. Activated CEC were identified based on the expression of adhesion or procoagulant molecules (CD54, CD62E and CD142). The absolute numbers of total CEC and CEP were calculated according with the BD Trucount™ tube manufacturer's recommendations. The absolute numbers of activated CEC were calculated by multiplying the absolute numbers of the total CEC by the percentage of activated CEC, for each marker in the study. As CEC are very rare events, all samples were acquired till the end.

### Statistics

Statistical analysis was performed using the SPSS statistical software (version 13.0). For each continuous quantitative variable, the median, minimum and maximum values were calculated. The Mann-Whitney test was used to compare absolute numbers of total CEC and CEP, as well as the absolute numbers of activated CEC between groups, whereas the Spearman's test was used to correlate variables. P values less than 0.05 were considered statistically significant.

## Results

### Clinical data and risk factors for thrombosis

The demographic and clinical data of patients and controls are presented in Table 2. The distribution of gender in patients with MPN and VTE did not differ significantly to that observed in controls. The VTE group was found to be similar to the control group concerning the distribution of age, but patients MPN were significantly older than controls (p = 0.001). Although there is not age match between patients with MPN and controls, we verified that the age is not associated with the variables of interest in our study, not having a confounding effect (Tables S1 and S2 in File S1).

**Table 2:** Demographic and clinical data of patients with VTE, patients with MPN and controls.

Demographic and clinical features		Controls	VTE	MPN	MPN (ET)	MPN (PV)
Number of cases		20	16	17	9	8
Gender (males: females)		10:10	4:12	9:8	2:7	7:1
Age (years)		49.5 (34–64)	38 (22–65)	64 (32–83)	57 (32–83)	64.5 (59–82)
Number of patients with TE, n (%)		0	16 (100.0)	5 (29.4)	2 (22.2)	3 (37.5)
Number of TE per patient		0 (0)	1 (1–4)	0 (0–1)	0 (0–1)	0 (0–1)
Type of TE, n (%)	Femoral and iliofemoral	0 (0)	14 (87.5)	1 (5.9)	0 (0)	1 (12.5)
	Pulmonary thromboembolism	0 (0)	1 (6.3)	0 (0)	0 (0)	0 (0)
	Venous TE at unusual sites	0 (0)	2 (12.5)	3 (17.6)	2 (22.2)	1 (12.5)
	Arterial thrombosis	0 (0)	1 (6.3)	1 (5.9)	0 (0)	1 (12.5)
Other clinical features, n (%)	Family history of thrombosis	0 (0)	2 (12.5)	NA	NA	NA
	Recurrent miscarriages	0 (0)	0 (0)	NA	NA	NA
Treatment, n (%)	No treatment	20 (100.0)	14 (87.5)	1 (5.9)	1 (11.1)	0 (0)
	Hydroxyurea	0 (0)	0 (0)	12 (70.6)	5 (55.6)	7 (87.5)
	Antiplatelet drugs	0 (0)	0 (0)	7 (41.2)	6 (66.7)	1 (12.5)
	Phlebotomies	0 (0)	0 (0)	5 (29.4)	0 (0)	5 (62.5)
	Oral anticoagulants	0 (0)	1 (6.3)	2 (11.8)	0 (0)	2 (25.0)
	Low molecular weight heparins	0 (0)	1 (6.3)	0 (0)	0 (0)	0 (0)

Abbreviations: ET, essential thrombocythaemia, VTE, venous thromboembolism; MPN, myeloproliferative neoplasms; PV, polycythaemia vera; TE, thromboembolic events; NA, data not available.

Results are presented as median (minimum – maximum) values and as absolute (n) or relative (%) frequencies.

In the VTE group the median number of TE was 1 (range: 1–4), while in the MPN group the median was 0 (range: 0–1) (Table 2). Of the 17 patients with MPN, 5 had thrombosis, belonging 2 to the ET subgroup and 3 to the PV subgroup. We did not find any correlation between gender and age with the number and the type of TE (data not shown). At the time of the study, 2 patients with VTE and 16 patients with MPN (8 ET patients and 8 PV patients) were receiving therapy.

Patients with VTE had significantly lower haematocrit values than controls (Table 3), as previously described [28]. On the other hand, patients with MPN had a significantly higher number of platelets and white blood cells (WBC) when compared to controls; similar results were obtained when the ET and PV subgroups were analyzed separately. Moreover, patients with PV had significantly higher haemoglobin and haematocrit values than controls. We did not find any correlation between the WBC, platelets, haematocrit and haemoglobin values and the number of TE in MPN group, neither in the ET and PV subgroups (data not shown). No VTE patients had the Factor V Leiden mutation but one patient was heterozygous for the prothrombin gene G20210 G->A mutation. The Jak2 mutation was detected in 11 patients of the patients with MPN (61.1%), 5 patients with ET (55.6%) and 6 patients with PV (75.0%). We did not observe any correlation between the blood counts and the presence of Jak2 mutation, neither in the MPN group as an all, nor in the ET and in the PV subgroups. In the VTE group there were several patients with high levels of fibrinogen, factor VIII, D-dimers and homocysteine and/or with low levels of free protein S and protein C (Table 4). Nevertheless there was no correlation between these parameters and the number of thrombosis in VTE patients.

**Table 3:** Blood cell counts in patients with VTE, patients with MPN and controls.

Blood counts	Controls	VTE	P*	MPN	P*	MPN (ET)	P*	MPN (PV)	P*
HGB (g/dl)	14.1 (11.8–15.8)	13.5 (11.7–16.2)	NS	14.5 (11.1–16.6)	NS	13.5 (11.1–15.6)	NS	14.8 (13.8–16.6)	0.021
HT (%)	42.1 (36.2–47.2)	39.6 (35.2–48.3)	0.042	44.0 (34.7–49.8)	NS	40.5 (34.7–47.1)	NS	46.2 (43.4–49.8)	<0.001
PLT (x10^9^/l)	215 (149–306)	216.5 (169–364)	NS	635 (154–1033)	<0.001	705 (567–1033)	<0.001	406 (154–1026)	0.011
WBC (x10^9^/l)	6.5 (3.9–11.2)	6.8 (4.2–10.1)	NS	11.6 (7.1–24.7)	<0.001	11.6 (7.3–24.7)	<0.001	10.4 (7.1–23.6)	<0.001

Abbreviations: ET, essential thrombocythaemia; VTE, venous thromboembolism; MPN, myeloproliferative neoplasms; PV, polycythaemia vera; HGB, haemoglobin; HT, haematocrit; PLT, platelets; WBC, white blood cells; NS, not statistically significant.

Results are presented as median (minimum – maximum) values.

* Patients *versus* controls; Mann-Whitney test; p<0.05 was considered statistically significant.

**Table 4:** Other risk factors in patients with VTE.

	Values in patients with VTE	Reference values	Number of patients with VTE with abnormal values (%)
Fibrinogen (g/l)	3.0 (2.1–5.0)	2.2–3.8	7 (43.8)
Factor VIIIc (%)	155.3 (74.0–245.6)	60–170	6 (37.5)
Homocysteine (µmol/l)	8.5 (4.8–31.0)	<15	1 (6.3)
D-dimers (ng/ml)	141.5 (36.0–854.0)	<500	2 (12.5)
Free protein S (%)	89.4 (52.4–115.2)	F: >55; M: >65	1 (6.3)
Protein C (%)	102.4 (54.0–150.0)	>70	1 (6.3)
Antithrombin (%)	108.0 (89.0–133.0)	>80	0 (0)
Anti-β2-GPI, IgM (MPL/ml)	3,5 (0.5–12.2)	<18	0 (0)
Anti-β2-GPI, IgG (GPL/ml)	2.1 (0.7–17.6)	<18	0 (0)
ACA, IgM (MPL/ml)	1.0 (0.0–13.3)	<15	0 (0)
ACA, IgG (GPL/ml)	2.0 (0.0–39.0)	<15	0 (0)
Lupus anticoagulant (ratio)	1.0 (0.8–1.2)	<1.2	0 (0)

Abbreviations: VTE, venous thromboembolism; ACA, anti-cardiolipin antibodies; anti-β2-GPI, anti-beta2 glycoprotein I antibodies; F, females; M, males.

Results are presented as median (minimum – maximum) values and as absolute (n) or relative (%) frequencies.

### Circulating endothelial cells

The median count of CEC in healthy individuals was 504 cells/ml, ranging from 186 to 1371 (Table 5). Both patient groups had a statistically significant increase in the CEC numbers, as compared to controls. The highest CEC numbers were observed in MPN patients (median of 1305 cells/ml; range: 499–4616), while VTE patients had intermediate numbers of CEC (median of 1231 cells/ml, range: 446–4272). In addition, patients with ET had higher numbers of CEC (1305 cells/ml, range: 499–4239) than PV patients (1231 cells/ml, range: 594–4616).

**Table 5:** Absolute numbers of CEC and CEP in patients with VTE, patients with MPN, and controls.

Group	CEC/ml	P*	CEP/ml	P*
Control	504 (186–1371)	NA	295 (0–2110)	NA
VTE	1231 (446–4272)	<0.001	168 (0–1698)	0.029
MPN	1305 (499–4616)	<0.001	311 (0–3791)	NS
MPN (ET)	1305 (499–4239)	0.001	311 (83–2283)	NS
MPN (PV)	1231 (594–4616)	0.001	385 (0–3791)	NS

Abbreviations: CEC, circulating endothelial cells; CEP, circulating endothelial progenitor cells; MPN, myeloproliferative neoplasms; VTE, venous thromboembolism; ET, essential thrombocythaemia, PV, polycythaemia vera; NA, not applicable; NS, not statistically significant.

Results are presented as median (minimum – maximum) values.

* Patients *versus* controls; Mann-Whitney test; p<0.05 was considered statistically significant.

### Circulating endothelial progenitor cells

The median count of CEP in healthy individuals was 295 cells/ml, ranging from 0 to 2110 (Table 5). The number of CEP in patients with MPN (311 cells/ml, ranging from 0 to 3791) was higher than that observed in controls, although differences were not statistically significant. Similar results were obtained when the ET and PV subgroups were analysed separately. In contrast, patients with VTE had a lower CEP count than controls (168 cells/ml; range 0–1698) (p<0.05).

### Activated circulating endothelial cells

Activated CEC were identified based on the expression of adhesion (CD54, CD62E) or procoagulant (CD142) molecules (Table 6).

**Table 6:** Absolute numbers of CEC expressing activation-related adhesion and procoagulant molecules.

Groups	CD54+ CEC/ml	P*	CD62E+ CEC/ml	P*	CD142+ CEC/ml	P*
Control	72 (0–227)	NA	145 (0–604)	NA	77 (0–366)	NA
VTE	238 (29–1179)	<0.001	683 (205–2990)	<0.001	309 (93–2532)	<0.001
MPN	140 (0–521)	NS	677 (200–2390)	<0.001	0 (0–1154)	NS
MPN (ET)	140 (0–493)	NS	696 (200–1941)	<0.001	0 (0–618)	NS
MPN (PV)	124 (0–521)	NS	602 (359–2390)	<0.001	47 (0–1154)	NS

Abbreviations: CEC, circulating endothelial cells; MPN, myeloproliferative neoplasms; VTE, venous thromboembolism; ET, essential thrombocythaemia, PV, polycythaemia vera; NA, not applicable; NS, not statistically significant.

Results are presented as median (minimum – maximum) values.

* Patients *versus* controls; Mann-Whitney test; p<0.05 was considered statistically significant.

All patient groups had higher numbers of CD62E+ CEC, as compared to controls (145 cells/ml, ranging from 0 to 604) (p<0.001). The median count of CD62E+ CEC in VTE patients was 683 cells/ml, ranging from 205 to 2990, similar values were observed in patients with MPN (677 cells/ml, ranging from 200 to 2390).

We also notice that all groups had higher numbers of CD54+ CEC as compared to healthy individuals, who had a median number of CD54+ CEC of 72 cells/ml, ranging from 0 to 227. However differences reached statistical significance only for VTE patients (p<0.001), who showed a median number of CD54+ CEC of 238 cells/ml, ranging from 29 to 1179.

In that concerning the expression of the CD142 procoagulant marker (TF), we found that only VTE patients had significantly higher numbers of CD142+ CEC (median: 309 cells/ml; range 93–2532) than healthy control (median: 77 cells/ml; range: 0–366) (p<0.001), no differences being observed between the CD142+ CEC counts from patients with MPN (ET or PV) and controls.

### Correlation between total circulating endothelial cells, activated endothelial cells and endothelial cell precursors with other clinical and laboratory data

In VTE and MPN groups we found a positive correlation between the total number of CEC and the WBC count (VTE: r = 0.515, p = 0.041; MPN: r = 0.738, p = 0.001) and between the number of CD62E+ CEC and the WBC count (VTE: r = 0.605, p = 0.013; MPN: r = 0.610, p = 0.009). Moreover, patients with MPN also had a positive correlation between the number of CEP and the WBC count (r = 0.846, p<0.001) and a negative correlation between the CD54+ CEC and the platelet count (r = −0.508, p = 0.037). We did not find any correlation between the number of total and activated CEC and CEP and the presence of Jak2 mutation in MPN patients (data not shown).

In patients with VTE, we observed that the number of CD142+ CEC correlated with the number of thrombosis (r = 0.568, p = 0.022). When investigating the correlation between total CEC, total CEP and activated CEC counts and other thrombophilia factors, we found that the number of CD54+ CEC correlated positively with plasma level of AT (r = 0.558, p = 0.025).

## Discussion

The identification and quantification of the EC in the blood is technically very difficult and not yet well standardized [13], which probably explains the wide range of CEC numbers described in healthy individuals (4 to 7900 cells/ml) [10], [29]. Nevertheless, efforts have been made to define correctly the CEC, as well as the CEP, and there is evidence that these two EC populations can reveal endothelial injury and endothelial repair, respectively. CEC are thought to be released from the vessel wall after vascular damage, while CEP are derived from the bone marrow and mobilized to injury sites in order to assist in the vascular repair [7], [8], [11], [30], [31] In healthy individuals there is a balance between CEC and CEP that preserves cell integrity and homeostasis [32], this balance being disrupted in some pathological conditions, in which there are increased numbers of CEC and/or decreased numbers of CEP [7], [8], [9], [10], [31], [33], [34], [35], [36]. Moreover, it has been found that increased numbers of CEC were accompanied by alterations of endothelial soluble markers [37]. Thus, it seems reasonable to assume that the simultaneous study of CEC and CEP can reveal endothelial state and actual function [11], [32], [38].

In this study we challenged a new approach to analyzing the CEC and CEP, using a four-color staining lyse-no-wash flow cytometry procedure together with specific acquisition and analysis strategies. Using this procedure, we identified the CEC and the CEP based on the differential expression of the CD45, CD146 and CD133 molecules. In accordance, CD146 is widely used to recognize CEC, and also identifies CEP [39]. Although T-cells may also express CD146, using CD45, a pan leukocyte marker, it is possible to discriminate CD45+highCD146+ T-cells from CEC, which are CD45-CD146+, and from CEP, which are CD45+lowCD146+ [13]. In addition, CD133, a progenitor molecule expressed on the CEP and haematopoietic stem cells, is absent in CEC [39], [40], [41]. Therefore, co-staining for CD146, CD133 and CD45, allows to distinguish between CEP (CD45+lowCD146+CD133+), CEC (CD45-CD146+CD33-), and other blood cell types [39].

During acquisition we gated both CD45-CD146+ and CD45+CD146+ events, which include CD146+ T-cells, the identification of CEC being made only in the analysis step; this gave the advantage of using activated T-cells to define the cut-off for the expression of CD146 [42].

To the best of our knowledge, this is the first report in which CEC and CEP were simultaneously quantified and, at the same time, the CEC were characterized for the expression of the activation (CD62E, CD54) and procoagulant (CD142) molecules in patients with VTE and MPN. In that concerning the MPN group, it is important to mention that most results from patients with ET and PV were presented together because these subgroups were small when considered individually and we did not find significant statistic differences between them in most parameters.

In the present study, the median CEC counts in the control group was 504 cells/ml, which is in the same range of values found by another group [43]. Our results also showed that CEC numbers are significantly higher in patients with VTE and MPN, as compared to healthy individuals. Increased numbers of CEC in patients with ET and PV have already been reported in a previous study [29].

Of note, we found significantly lower numbers of CEP in patients with VTE in comparison to those observed in controls. Since there is evidence that CEP promote new vessel formation and tissue vascularisation during ischemia [44], this observation may indicate impaired endothelial repair in patients with VTE. On the other hand, MPN patients showed higher numbers of CEP, as compared to controls, although the difference was not statistically significant. This finding is consistent with a previous report showing higher numbers of CEP in the PV patients [45] and could explain the increased microvessel density described in patients with MPN [46].

Concerning the expression of activation- and procoagulant-related molecules, we found that all disease groups analysed in this study had higher counts of CD62+ CEC, as compared to controls. On the contrary, only VTE patients showed significantly higher CD54+ and CD142+ CEC numbers, suggesting that adhesion molecules are involved in the vascular occlusion in these patients.

When correlating total- and activated-CEC numbers with blood counts, we found a positive correlation between the WBC and the CEC, as well as between the WBC and the CD62E+ CEC counts, in both disease groups. The stronger and more significant correlation observed in the MPN group, may indirectly indicate the endothelial involvement in thrombosis, since it was previously reported that leukocytosis is associated with thrombosis in patients with MPN [47], [48]. In addition, a positive correlation between the WBC count and soluble CD62E was found in another study [49]. Our results are in conformity with the presence of inflammation, as the WBC count is an inflammatory marker [50], being accompanied with increased numbers of total and activated CEC.

In patients with MPN we also found a positive correlation between the CEP and the WBC counts. It is well known that MPN are stem cell disorders, suggesting that is accompanied also by an increase in EC [51]. According to a previous study, there is evidence that EC from patients with MPN originate from the same pathological clone as the hematopoietic cells, since these cells have a same common precursor, the hemangioblast [51].

Moreover, in MPN patients we found a negative correlation between the platelet and the CD54+ CEC counts. Maybe the fact that most patients with MPN were on therapy had influenced the number of platelets since it is already well established the ability of hydroxyurea to reduce the platelet count [52], and to increase the membrane-bound ICAM-1 [53].

Regarding to VTE patients, we found a positive correlation between the number of CD54+ CEC and the AT plasma level. It has been known that AT has anti-inflammatory activity, besides its role in inhibiting thrombin and other coagulation enzymes. In fact, several authors, studying different models, made the observation that AT reduces the leukocyte adhesion and inflammation [54], [55], [56]. Moreover, it was also shown that AT suppresses platelet adhesion onto immobilized fibrinogen under low flow condition [57]. The mechanism by which AT exerts these effects in leukocyte and platelet adhesion and in ICAM-1 is still unknown, but it was reported that AT administration increases the levels of prostacyclin (PGI2), which may explain at least in part its anti-inflammatory properties [58]. Considering these results, at first sight it could be expected a negative correlation between the numbers of CD54+ CEC and the levels of AT, curiously that was not what we observed. Nevertheless, it should be noted that none of these studies were done in VTE patients, but rather in induced inflammatory models.

In VTE patients, there was a positive correlation between the number of CD142+ CEC and the number of TE. This finding is not surprising, as TF is a key indicator of the coagulation activation [59], and previous studies in animal models have shown that this molecule has an important role in thrombosis development [60], [61]

In conclusion, our results demonstrated that CEC numbers are increased in patients with VTE and MPN, revealing endothelial injury. In addition, the lower numbers of CEP detected in VTE, in comparison to controls and MPN patients, would suggest a deficiency on vascular repair. Our data also indicate that the activation (CD54 and CD62E) and procoagulant (CD142) related molecules are differentially expressed on CEC from patients with VTE and MPN. It is also worth of note that total and activated-CEC numbers and blood counts correlate differently in each patient group. In addition, in patients with VTE we found a positive correlation between the number of CD54+ CEC and the AT levels, whereas the number of CD142+ CEC correlated positively with the number of thrombosis. Altogether, the results presented here reinforce the use of CEC and CEP as vascular biomarkers and seem to indicate that those cells may have a role in the mechanism involved in thrombosis, which appears to be different in VTE and MPN patients.

## Supporting Information

File S1 **Contains: Table S1**. Correlation between age and the number of the EC populations in the blood of patients with VTE. **Table S2**. Correlation between age and the number of the EC populations in the blood of patients with MPN.
(TIF)
