# Supplementary figures and images for: Circulating Endothelial Cells in Patients with Venous Thromboembolism and Myeloproliferative Neoplasms
Source: PLoS One. 2013 Dec 5;8(12):e81574. doi: 10.1371/journal.pone.0081574 (PMC3855326; doi:10.1371/journal.pone.0081574)

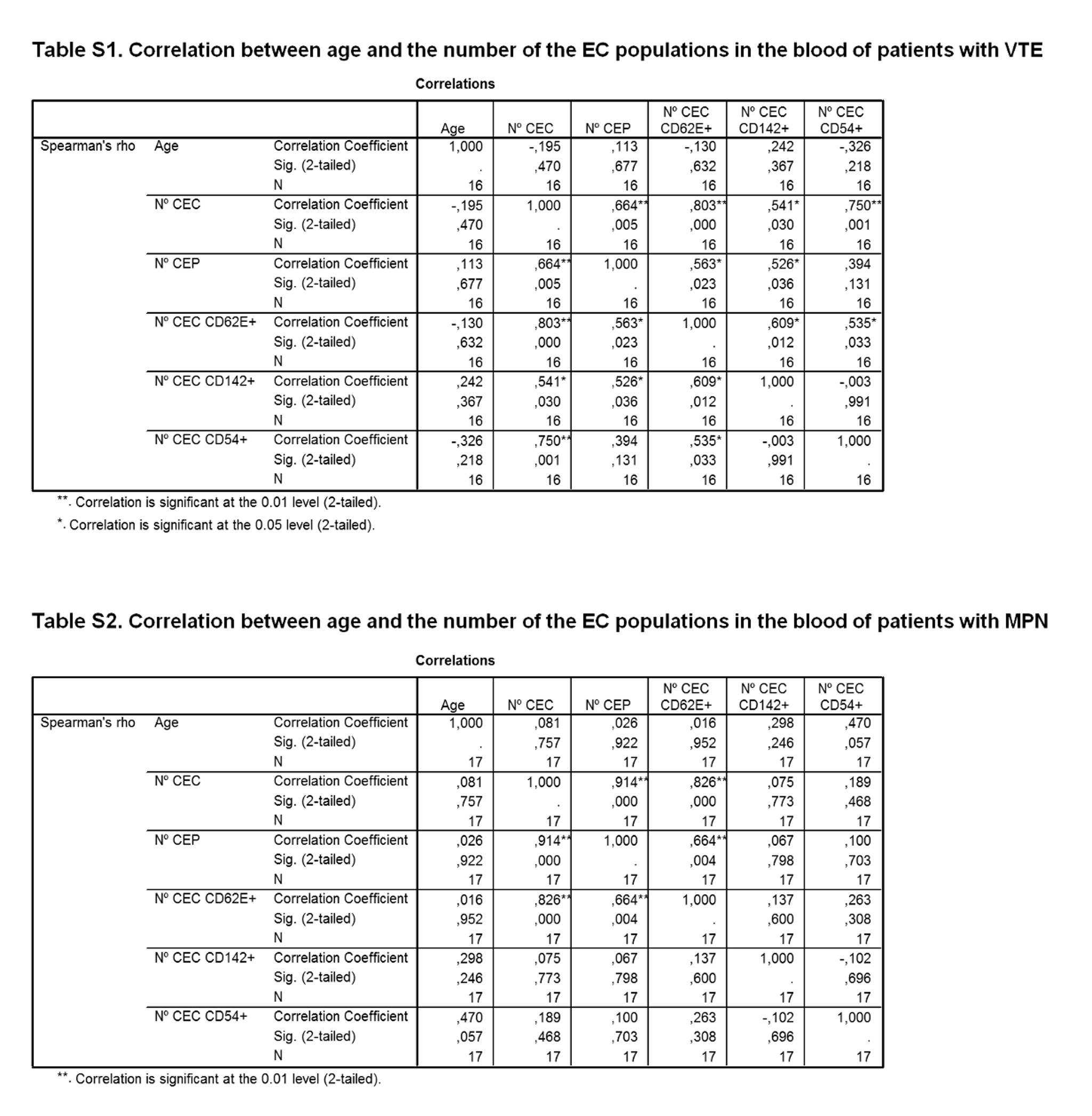

Supplement: File S1 — Contains: Table S1. Correlation between age and the number of the EC populations in the blood of patients with VTE. Table S2. Correlation between age and the number of the EC populations in the blood of patients with MPN. (TIF) [file pone.0081574.s001.tif]
